# Structural mechanism of helicase loading onto replication origin DNA by ORC-Cdc6

**DOI:** 10.1073/pnas.2006231117

**Published:** 2020-07-15

**Authors:** Zuanning Yuan, Sarah Schneider, Thomas Dodd, Alberto Riera, Lin Bai, Chunli Yan, Indiana Magdalou, Ivaylo Ivanov, Bruce Stillman, Huilin Li, Christian Speck

**Affiliations:** ^a^Structural Biology Program, Van Andel Institute, Grand Rapids, MI 49503;; ^b^DNA Replication Group, Institute of Clinical Sciences, Faculty of Medicine, Imperial College London, W12 0NN London, United Kingdom;; ^c^Department of Chemistry, Georgia State University, Atlanta, GA 30302;; ^d^Center for Diagnostics and Therapeutics, Georgia State University, Atlanta, GA 30302;; ^e^Cold Spring Harbor Laboratory, Cold Spring Harbor, NY 11724;; ^f^MRC London Institute of Medical Sciences (LMS), W12 0NN London, United Kingdom

**Keywords:** DNA replication, cryo-EM, Mcm2-7 helicase, ATPase, origin recognition complex

## Abstract

DNA replication origins serve as sites of replicative helicase loading. In all eukaryotes, the six-subunit origin recognition complex (Orc1-6; ORC) recognizes the replication origin. During late M-phase of the cell-cycle, Cdc6 binds to ORC and the ORC–Cdc6 complex loads in a multistep reaction and, with the help of Cdt1, the core Mcm2-7 helicase onto DNA. A key intermediate is the ORC–Cdc6–Cdt1–Mcm2-7 (OCCM) complex in which DNA has been already inserted into the central channel of Mcm2-7. Until now, it has been unclear how the origin DNA is guided by ORC–Cdc6 and inserted into the Mcm2-7 hexamer. Here, we truncated the C-terminal winged-helix-domain (WHD) of Mcm6 to slow down the loading reaction, thereby capturing two loading intermediates prior to DNA insertion in budding yeast. In “semi-attached OCCM,” the Mcm3 and Mcm7 WHDs latch onto ORC–Cdc6 while the main body of the Mcm2-7 hexamer is not connected. In “pre-insertion OCCM,” the main body of Mcm2-7 docks onto ORC–Cdc6, and the origin DNA is bent and positioned adjacent to the open DNA entry gate, poised for insertion, at the Mcm2–Mcm5 interface. We used molecular simulations to reveal the dynamic transition from preloading conformers to the loaded conformers in which the loading of Mcm2-7 on DNA is complete and the DNA entry gate is fully closed. Our work provides multiple molecular insights into a key event of eukaryotic DNA replication.

## Significance

The loading of the core Mcm2-7 helicase onto origin DNA is essential for the formation of replication forks and genomic stability. Here, we report two cryo-electron microscopy (cryo-EM) structures that capture helicase loader–helicase complexes just prior to DNA insertion. These pre-loading structures, combined with a computational simulation of the dynamic transition from the pre-loading state to the loaded state, provide crucial insights into the mechanism required for topologically linking the helicase to DNA. The helicase loading system is highly conserved from yeast to human, which means that the molecular principles described here for the yeast system are likely applicable to the human system.

DNA replication is central for cellular life and genomic stability. Most macromolecular processes are controlled at the level of initiation (1). In eukaryotes, DNA replication is separated into two distinctive steps occurring in different cell cycle phases (2). During G1 phase of the cell cycle, the helicase core consisting of the minichromosome-maintenance 2–7 (Mcm2-7) proteins becomes loaded onto DNA, a process termed prereplicative complex (pre-RC) formation. Then, in S phase, the Mcm2-7 hexamer becomes activated, leading to origin firing and DNA synthesis. Helicase loading at DNA replication origins depends on the Origin Recognition Complex (ORC), Cdc6, and Cdt1 (3–7). Yeast ORC binds the replication origin DNA and is associated with DNA through the cell cycle (8, 9). During late M-phase, Cdc6 binds to ORC, transforming it into an active complex, which is now competent to load, with the help of Cdt1, two Mcm2-7 hexamers into Mcm2-7 double hexamer that encircles DNA (6, 7). Prior to helicase loading, the Mcm2-7 single hexamer adopts an open spiral configuration (10–12). However, within the ORC–Cdc6–Cdt1–Mcm2-7 (OCCM) pre-RC intermediate, which forms in the absence of ATP hydrolysis, DNA is already inserted into Mcm2-7 and the helicase adopts a ring-shaped conformation, with a small gap at the Mcm2–Mcm5 interface (13–17). Based on a recent reanalysis of the wild-type (WT) cryo-EM dataset (18), we were able to separate a minor population of OCCM in which the DNA is fully inserted and the Mcm2–Mcm5 gate is fully closed at both N- and C-tiers. To facilitate the description of the structural transition in this report, we refer to these two WT OCCM conformers as “OCCM” (gate partially closed) and “gate-closed OCCM” (gate fully closed), respectively.

Upon ATP hydrolysis, Cdc6 and Cdt1 are released from the OCCM and a second Mcm2-7 hexamer is recruited to the origin, resulting in an Mcm2-7 double-hexamer, with both hexamers connected via their N-termini and the ring fully closed (6, 12, 13, 19–23). Whether the same ORC or a different ORC loads the second Mcm2-7 hexamer is still controversial (13, 19, 20, 24).

Orc1-5 and Cdc6 belong to the AAA+ family of ATPases (25, 26), while Orc6 shares homology with the transcription factor II B (TFIIB) (27, 28). Orc1, Orc4, Orc5, and Cdc6 participate in ATP binding and are composed of an AAA+ domain followed by a winged-helix domain (WHD), while Orc2 and Orc3 do not bind to ATP and are structurally more diverged (29). In particular, Orc3 is characterized by a large α-helical insertion located between its AAA+ domain and the WHD, giving rise to the typical asymmetric shape of the ORC complex (30, 31). More recent structural work revealed that ORC adopts a two-tiered hexameric structure, with one narrow tier made up by five Orc1-5 WHDs and one wider tier composed of the five AAA+ domains (18, 32–34). Orc6 is attached to the side of the ORC complex via Orc3, but also has major interactions with Orc2 and Orc5 (18, 33, 35). When ORC is DNA-bound, DNA passes through its central channel. On the narrow C-terminal side of the complex, Orc3, Orc5, and Orc6 bend the DNA by 60 ° utilizing patches of positively charged amino acids (33).

Biochemical and genetic work has shown that the Mcm3 C-terminal WHD is essential to promote initial Cdt1–Mcm2-7 interactions with ORC–Cdc6 (15). Indeed, point mutants in the Mcm3 WHD have been shown to abrogate complex formation. In addition, the Mcm3 WHD is sufficient to interact with ORC–Cdc6, and so it was proposed that Mcm3 could initiate Mcm2-7 loading on DNA (15). Moreover, it was shown that a Cdt1–Mcm6 interaction remodels the Mcm2-7 complex, relieving an autoinhibitory activity of the C-terminal WHD of Mcm6. In budding yeast, this remodeling is critical for the initial ORC–Cdc6 interaction with Cdt1–Mcm2-7 (14). In metazoans, where Cdt1 and Cdc6 bind directly to ORC (36, 37), an ORC–Cdc6–Cdt1 complex may regulate the recruitment of Mcm2-7 via a Cdt1–Mcm6 interaction.

To study the initial phase in ORC–Cdc6–Cdt1–Mcm2-7 complex formation, it is necessary to slow down the loading process. Here we employed a previously reported Mcm6 mutant, which is missing the C-terminal WHD (amino acids 839 to 1017) and is referred to as McmΔC6 or “mutant Mcm2-7” (14). This mutant Mcm2-7 supports the first step in helicase recruitment, the interaction between Cdt1–Mcm2-7 and ORC–Cdc6, but fails to induce pre-RC ATP hydrolysis for unknown reasons and does not allow double hexamer formation (14). Thus, pre-RC formation is blocked at a stage prior to full OCCM complex formation. Using cryo-EM, we have now captured two OCCM intermediates, “semi-attached OCCM” and “pre-insertion OCCM,” which reveal the step-wise recruitment of budding yeast Cdt1–Mcm2-7 to ORC–Cdc6. Furthermore, to understand the dynamic DNA insertion process, we performed molecular dynamics simulations, revealing the energy landscape and the large conformational changes accompanying origin DNA insertion into Mcm2-7. Our work explains why ORC alone in the absence of Cdc6 is incapable of recruiting Cdt1-bound Mcm2-7, thereby demonstrating how ORC–Cdc6 guides and positions the DNA near the DNA entry gate in Mcm2-7 prior to helicase loading and how DNA enters into the Mcm2-7, providing mechanistic insights into a key stage of the highly conserved eukaryotic helicase-loading process.

## Results and Discussion

### Cryo-EM Captures Two OCCM Intermediates Using the Mcm6-ΔC6 Mutant.

**Fig. 1.**
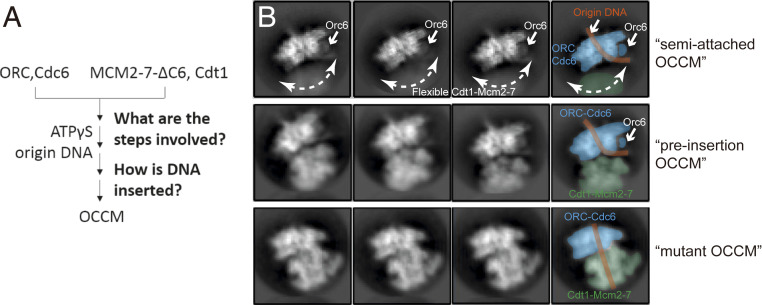
Cryo-EM of the origin DNA loading reaction using ORC, Cdc6, Cdt1, and a mutant Mcm2-7. (*A*) A sketch for the experimental procedure. (*B*) Two-dimensional averages of three distinct conformations: semi-attached OCCM, pre-insertion OCCM, and mutant OCCM. The approximate range of flexibility observed between ORC–Cdc6 and Cdt1-Mcm2-7 of the semi-attached OCCM is indicated with double-headed dashed curves.

To examine the structural rearrangements occurring during helicase loading in budding yeast, we employed an Mcm2-7 complex containing McmΔC6 and the ATP analog ATPγS, which both slow down the pre-RC reaction cascade (Fig. 1 *A*) (14). Analysis of 2D class averages showed one set of classes where the density of the helicase loader ORC–Cdc6 is well resolved while Cdt1–Mcm2-7 is clearly present but is blurred (Fig. 1 *B*, *Upper*). This suggests the capture of a very early intermediate in which Mcm2-7 is connected to ORC–Cdc6 yet the two complexes have not docked onto each other. We termed this intermediate semi-attached OCCM. Further analysis of the 2D class averages revealed a second intermediate in which both ORC–Cdc6 and Cdt1–Mcm2-7 densities were well resolved, indicating that ORC–Cdc6 and Cdt1–Mcm2-7 have docked onto each other (Fig. 1 *B*, *Center*) (18). Interestingly, the origin DNA exiting from the ORC–Cdc6 complex is found in the cleft between ORC–Cdc6 and the top of the Mcm2-7 helicase core, clearly outside the Mcm2-7 central channel. Since this intermediate is prior to loading of Mcm2-7 on DNA, but more advanced than semi-attached OCCM, we have termed it “pre-insertion OCCM.”

We also observed 2D class averages in which origin DNA resides in the Mcm2-7 central channel, suggesting the presence of a loaded conformation (termed “mutant OCCM”) and that the removal of Mcm6 WHD did not completely block the DNA insertion (Fig. 1 *B*, *Lower*). During 3D classification, we further obtained a 3D map of the mutant Mcm2-7 hexamer in the presence of ATPγS at 7.7-Å resolution (*SI Appendix*, Table S1 and Figs. S1 and S2), which was similar to the reported 7 to 8-Å resolution maps of the WT yeast Mcm2-7 bound to ADP or AMPPNP (10), albeit missing the Mcm6 C terminus. Therefore, the removal of Mcm6 WHD did not alter the left-handed open spiral architecture of Mcm2-7 hexamer.

### Structure of the Semi-Attached OCCM: ORC Undergoes Structural Changes to Become an Active Helicase Loader.

**Fig. 2.**
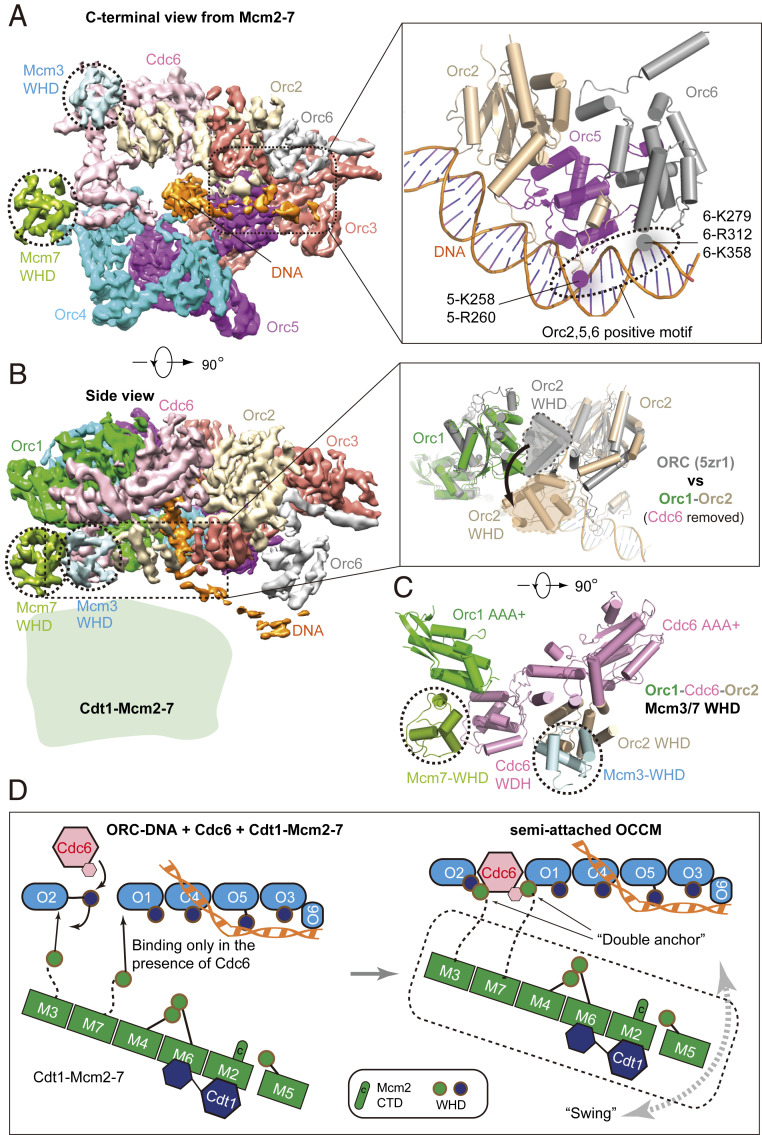
Cryo-EM structure of semi-attached OCCM. (*A*, *Left*) Cryo-EM 3D map viewed from the narrower, C-terminal tier. Subunits are individually colored. Orc1 has been omitted for clarity. (*Inset, Right*) ORC–DNA interaction outside the central channel. (*B*, *Left*) Side view of the 3D map. (*Inset, Right*) Orc2 WHD (shown in gray) occupies the gap between Orc1 and Orc2 in the ORC–DNA structure; this domain moves out (shown in beige) to accommodate Cdc6 binding in ORC–Cdc6–DNA. (*C*) The N-terminal view shows that Mcm3 WHD binds to the Orc2 WHD and the AAA+ domain of Cdc6 and Mcm7 WHD binds to the WDH of Cdc6 and the AAA+ domain of Orc1. (*D*) An open-book sketch showing that the binding interface for the Mcm3 and Mcm7 WHDs is not available in ORC–DNA and becomes available only when Cdc6 binds ORC–DNA. It also illustrates that the Cdt1–Mcm2-7 is largely flexible at the very first “anchoring” step.

Three-dimensional classification of the OCCM–ΔC6 dataset enabled us to identify a 4.3-Å average resolution structure of the semi-attached OCCM (*SI Appendix*, Table S1 and Figs. S1 and S3). In this complex, ORC and Cdc6 form a six-membered elongated ring with Cdc6 bridging the gap between Orc1 and Orc2. The ring is characterized by interdigitated domain-swapping interactions between the WHDs and the AAA+ domains of adjacent subunits to form a two-tiered ring. The density of ORC–Cdc6–DNA is nearly complete, but most of the Mcm2-7 density is missing, except for the Mcm3 and Mcm7 WHDs. The large AAA+ C-tier collar of ORC–Cdc6 is not obstructed and available for interaction with the C-terminal interface of the incoming Cdt1–Mcm2-7 (Fig. 2 *A* and *B*). From 2D classes, we can deduce a clear gap between the two main components, though the cloud-like appearance indicates Cdt1–Mcm2-7 is flexibly attached and displays a large degree of conformational flexibility relative to the helicase loader (Fig. 1*B*, dashed lines).

In the semi-attached OCCM, we observed the entire C-terminal domain of Orc6, which was making contacts with the origin DNA (Fig. 2*A*). This is in contrast to the previously published OCCM and the *Drosophila* ORC structures, in which only a single Orc6 α-helix was resolved and the Orc6–DNA interaction was absent (18, 38). Thus, the data strongly suggest that the C-terminal domain of Orc6 is stabilized by DNA, similar as in the ORC–DNA cryo-EM structure (33).

The ATPase subcomplex Orc1–Orc4–Orc5 in the semi-attached OCCM is largely unchanged when compared to the yeast ORC–DNA structure, except for the Orc2 WHD that is rotated and moved out to accommodate Cdc6 (Fig. 2 *B* and *C*). Therefore, the Orc2 WHD appears to serve as a flexible gate that allows DNA and Cdc6 insertion. This observation is consistent with the reported ORC–DNA structures in which the Orc2 WHD occupies different positions (33). Moreover, the Orc2 WHD appears to play a role in restricting DNA loss from the center of the ring, generating a topological link, which explains ORC’s strong affinity for DNA (39).

As mentioned above, in the semi-attached OCCM complex, DNA near the narrow C-terminal tier of the helicase loader is in a bent conformation, and is held in place by Orc6. The bent DNA corresponds to the B1-element of the *ARS1* origin and makes extensive contacts with Orc2, 5, and 6 via many positively charged and well-conserved residues similar to those in the ORC–DNA structure (33) (Fig. 2*A*). Moreover, in the semi-attached OCCM, the Orc4 insertion helix (IH) is seen binding the DNA major groove (18, 33), but not the basic patch of Orc1 that interacts with the ARS consensus sequence (ACS) (33, 40) (Fig. 2*A*). The Orc4 insertion loop, the sole connection between ORC–Cdc6 and Cdt1 as observed in the WT OCCM (18), was invisible in the semi-attached OCCM. This indicates that the Orc4 loop is flexible before it binds in an Mcm6 WHD-dependent manner to Cdt1 and that Orc4, Cdt1, and Mcm6 WHD form an intricate structural network.

### The Mcm3 and Mcm7 WHDs Act as a Double-Anchor in the First Rendezvous between Cdt1–Mcm2-7 and DNA-Bound ORC–Cdc6.

To our surprise, in addition to the well-defined ORC–Cdc6 density, we found two additional densities that were well resolved in the semi-attached OCCM (Fig. 2 *A*–*C*). In this structure, the Mcm3 WHD binds to the interface of the Orc2 WHD and Cdc6 AAA+ domain, and the Mcm7 WHD binds to the interface of the Cdc6 WHD and the Orc1 AAA+ domain (Fig. 2 *B* and *C*). The interaction surface of the Mcm3 and Mcm7 WHDs with Orc2–Cdc6 and Cdc6–Orc1, respectively, is similar to what has been observed in the WT OCCM (18), but the Mcm7 WHD adopts a slightly different conformation than in the WT OCCM. Interestingly, the Orc2–Cdc6 interface is the most dynamic region of the ORC–Cdc6 complex, as it controls DNA insertion and retention. Thus, Mcm3 and Mcm7 WHDs making contact with this interface suggest that they play a role in quality control to ensure that Mcm2-7 only engages with a fully assembled ORC–Cdc6–DNA complex. In other words, Cdt1–Mcm2-7 is unable to bind to ORC in the absence of Cdc6, explaining the absolute requirement of Cdc6 for Mcm2-7 loading (3, 6, 7, 41). Furthermore, by binding at the ORC–DNA gate, the Mcm3 and Mcm7 WHDs trap DNA in ORC–Cdc6, thereby stabilizing the complex, and in this way advancing pre-RC formation. These findings are consistent with a previous report, which showed that Mcm3 WHD regulates ORC–Cdc6 ATP hydrolysis and is essential for recruitment of Cdt1–Mcm2-7 to ORC–Cdc6 (15). Overall, the semi-attached OCCM structure suggests that, during the initial binding, Mcm2-7 first projects a double-anchor—the Mcm3 and Mcm7 WHDs—onto the ORC–Cdc6 platform (Fig. 2*D*). Thus, this intermediate may represent the earliest encounter between ORC–Cdc6 and Cdt1–Mcm2-7.

### Structure of the “Pre-Insertion OCCM”.

**Fig. 3.**
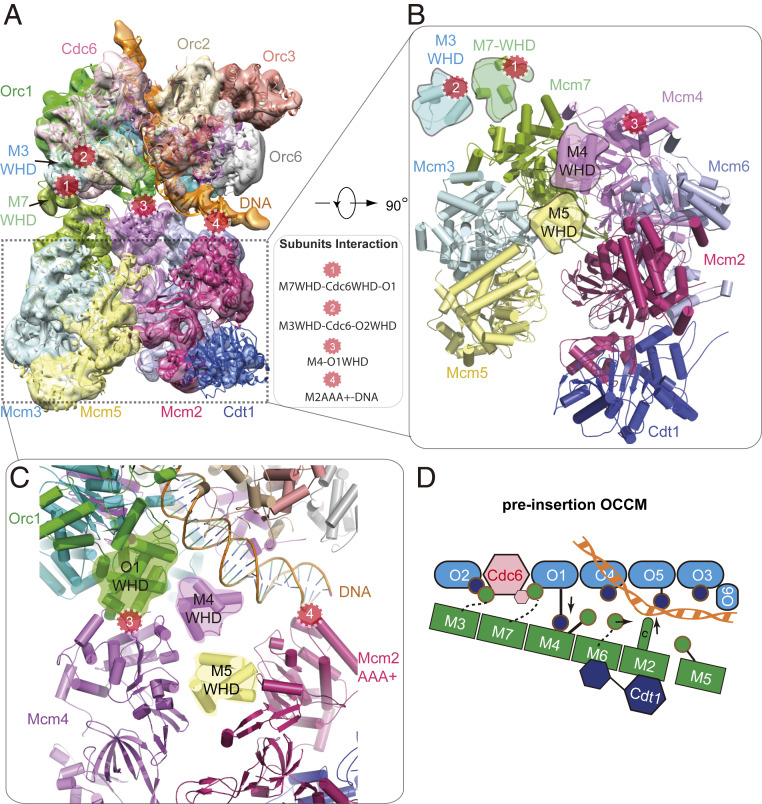
Cryo-EM structure of pre-insertion OCCM. (*A*) Cryo-EM 3D map in a side view, fitted with atomic models of ORC–Cdc6–DNA and the Cdt1-bound open spiral Mcm2-7. The four major contact sites between ORC-Cdc6 and Cdt1–Mcm2-7 are marked by four numbered red circles. (*B*) View of the Cdt1–Mcm2-7 in pre-insertion OCCM. Both Mcm4 and Mcm5 WHDs are on the top surface, preventing the full engagement of Mcm2-7 with ORC–Cdc6. (*C*) Zoomed view showing the contact sites 3 and 4. At site 3, Mcm4 WHD moves away, enabling Orc1 WHD moves in to interact with the AAA+ domain of Mcm4. At site 4, Mcm2 AAA+ domain interacts with and stabilizes DNA. (*D*) Sketch depicting the structural changes and interactions between Mcm4, 6, and 2 with Orc1 and DNA in pre-insertion OCCM.

Another helicase loading intermediate could be resolved, yielding an 8.1-Å resolution 3D map of the “pre-insertion OCCM” (Fig. 3 *A*–*D* and *SI Appendix*, Table S1 and Figs. S1 and S4). The atomic model contains all 14 pre-RC polypeptides, with DNA being clamped between ORC–Cdc6 and Cdt1–Mcm2-7, just prior to DNA insertion into the Mcm2-7 ring. In this intermediate, ORC–Cdc6 has a conformation similar to that in the semi-attached OCCM. This indicates that ORC–Cdc6 complex formation already preconfigures the helicase loader to accept Cdt1–Mcm2-7 for its initial recruitment. The mutant Cdt1–Mcm2-7 adopts a left-handed open spiral conformation, similar to WT Cdt1–Mcm2-7 (Fig. 3 *A* and *B*) (10). The B1-element of ARS origin DNA, just outside of the ORC–Cdc6 central channel, is bent by 60 ° and sandwiched between ORC and Mcm2-7. Crucially, at this stage of complex formation, the Mcm2–Mcm5 gate is open. However, the width of this gate is smaller than the 2-nm diameter of the DNA, suggesting that the gate needs to be widened when DNA is inserted through the Mcm2–Mcm5 gate.

The pre-insertion OCCM is characterized by a large wedge-shaped gap between ORC–Cdc6 and Cdt1–Mcm2-7, with Mcm4, Mcm6, and Mcm7 being in closest proximity to ORC–Cdc6 and Mcm3, Mcm5, and Mcm2 the furthest away. Four separate contact sites stabilize the ORC–Cdc6 interaction with Cdt1–Mcm2-7. The Mcm3 and Mcm7 WHDs remain bound to Orc2–Cdc6 as in the semi-attached OCCM (contact sites 1 and 2 in Fig. 3*A*). It is noteworthy that, in this complex, the Mcm7 WHD is in direct contact with its AAA+ domain (Fig. 3 *A* and *B*). Importantly, the Orc1 WHD establishes close contact with the AAA+ core of Mcm4 (contact site 3). Furthermore, in the pre-insertion OCCM, ORC–Cdc6 positions the DNA adjacent to the Mcm2–Mcm5 DNA entry gate, while also supporting DNA contacts with the AAA+ domain of Mcm2 (contact site 4 in Fig. 3 *C* and *D*). Thus, DNA is poised for insertion into the central channel of Mcm2-7 via the open Mcm2 and Mcm5 gate. It has been well known, but not understood why, that, among all six Mcm proteins, only Mcm2 lacks a C-terminal WHD. This mystery is readily explained by the positioning of the origin DNA right above Mcm2 in the pre-insertion OCCM structure, as an Mcm2 WHD could get in the way of DNA as it enters the Mcm2–Mcm5 gate.

### Structural Transition from Pre-Insertion OCCM to the OCCM.

**Fig. 4.**
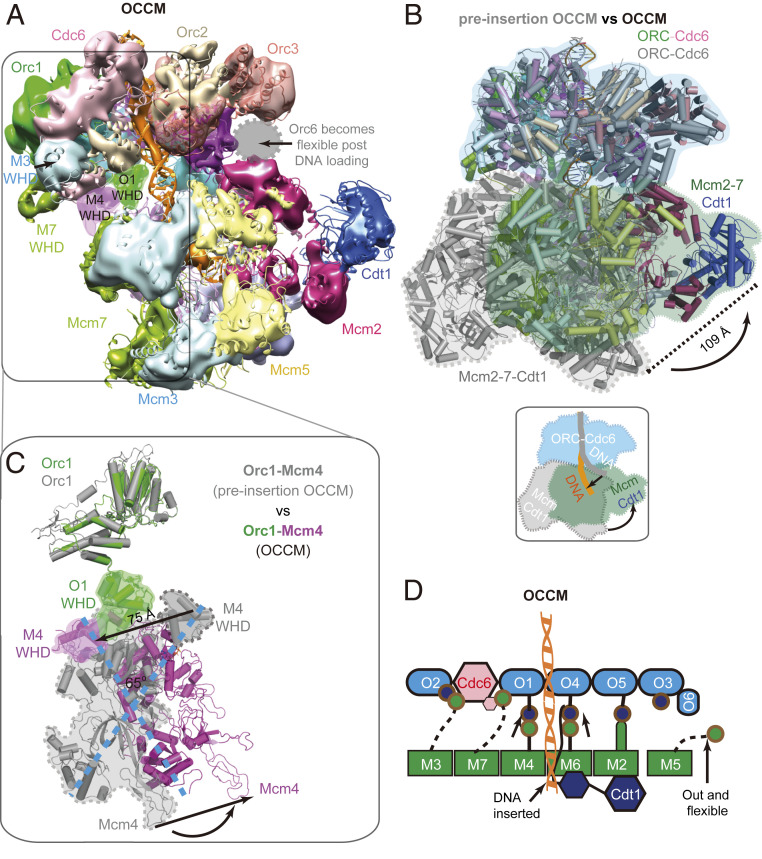
Conformational changes from pre-insertion OCCM to OCCM. (*A*) Side view of the cryo-EM 3D map of the mutant OCCM, rigid-body docked with the previously published WT OCCM structure (referred to as WT OCCM here). (*B*) Alignment of pre-insertion OCCM (gray) with OCCM (color) by superimposing their respective ORC–Cdc6 regions. (*C*) From pre-insertion OCCM (gray) to OCCM (color), Mcm4 rotates by ∼65 ° to interact with Orc1 with their respective WHDs. (*D*) Sketch of DNA configuration and interactions between ORC–Cdc6 and Mcm2-7 in the OCCM structure.

We previously demonstrated that deletion of the Mcm6 WHD supported ORC–Cdc6 interaction with Cdt1–Mcm2-7–ΔC6. However, Mcm2-7–ΔC6 is not competent to induce pre-RC ATP-hydrolysis or Mcm2-7 double hexamer formation (14). It has been unknown whether Mcm6 WHD is necessary to establish extensive ORC-Cdc6–Cdt1-Mcm2-7 interactions or DNA insertion. Now, 3D classification and 3D reconstruction of images of the OCCM-ΔC6 yielded a 3D map at 10.5-Å resolution of the mutant OCCM in which DNA was fully inserted in the Mcm2-7 channel (*SI Appendix*, Table S1 and Figs. S1 and S4). The previously reported 3.9-Å resolution structure of the WT OCCM aligns very well with the mutant 3D map (Fig. 4*A*) (18, 42). This implies that deletion of the Mcm6 WHD slowed down OCCM formation, but did not completely abolish the ORC-Cdc6–dependent insertion of DNA into Mcm2-7. During the transition from pre-insertion OCCM to WT OCCM, the main body of Mcm2-7 rotates and moves >100 Å to latch onto ORC–Cdc6 (Fig. 4*B*). In addition, Mcm2-7 switches from the spiral ring to a flat and largely closed ring conformation (18). Both the Mcm5 and Mcm4 WHDs clear the DNA channel, with Mcm5 WHD adopting a delocalized position and Mcm4 WHD moving by 75 Å to the outer rim of the complex to interact with Orc1 and Orc4 (Fig. 4*C*). In this way, Mcm4 and Mcm5 WHDs do not impede DNA insertion. Because the Mcm4 WHD is positioned within the central section of the pre-insertion OCCM and then moves out to interact with ORC in the OCCM, this sequence of events apparently imposes a directionality for Mcm2-7 loading (Fig. 4 *C* and *D*).

### Molecular Dynamic Simulation of DNA Insertion into the Mcm2-7.

Two major conformational changes are required during the transition from pre-insertion OCCM to the OCCM: DNA insertion through the Mcm2–Mcm5 gate and a switch of Mcm2-7 from an open spiral to a closed ring. One key event in this transition is the establishment of the interaction between Cdt1 and Mcm6, which is essential for pre-RC formation in yeast, mice, and humans (14, 43–45). Since the Mcm2–Mcm5 gate is not fully closed in the OCCM, there will also be significant changes in these two gate subunits as the structure transitions to the gate-closed OCCM, where the Mcm2–Mcm5 interface is fully engaged. To gain insight into the dynamic conformational changes accompanying these transitions, we employed state-of-the-art chain-of-replicas molecular simulation approaches. Specifically, we used the string method with swarms of trajectories (46) to compute an optimal path connecting the experimentally observed start state (pre-insertion OCCM) and the end state (gate-closed OCCM), with the OCCM as an experimental intermediate. The semi-attached OCCM was not used as the start point because the main body of Mcm2-7 was missing in that structure. The path was represented by 49 replicas of the simulation system, initially obtained by taking evenly spaced snapshots from a preliminary targeted molecular dynamics (MD) run. The path was then optimized by the string method on the 49 replicas in the space of two rmsd collective variables (CVs) as described in *Methods*.

**Fig. 5.**
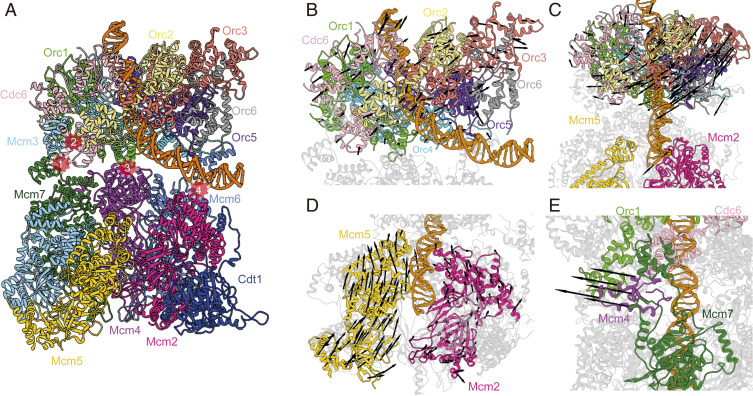
Key structural rearrangement along the partial nudged elastic band (PNEB) path reveals important global motions during pre-insertion OCCM–gate-closed OCCM. (*A*) Overall structure of pre-insertion OCCM. ORC–Mcm2-7 interactions are highlighted by red circles. (*B*) Global twist of ORC–Cdc6 with respect to Mcm2-7. Black arrows depict direction of twisting motion. Mcm2-7 is shown in gray while ORC–Cdc6 is colored according to *A*, *Inset*. (*C*) Global tilt of ORC–Cdc6 with respect to Mcm2-7. Black arrows depict direction of tilting motion. (*D*) Rearrangement of Mcm2 and Mcm5 subunits from open spiral to planar ring conformation. Black arrows depict direction of motion. (*E*) Mcm4–WHD as it transitions from inside to outside of OCCM. Black arrows only depict direction of motion for Mcm4–WHD.

The resultant minimum energy path (MEP) from the pre-insertion OCCM to the gate-closed OCCM shows a series of distinct motions (Fig. 5 *A*–*C* and Movie S1). ORC–Cdc6 first undergoes a tilt and a concomitant twist toward the Mcm2–Mcm5 gate (Fig. 5 *B* and *C*). Initially, ORC–Cdc6 and Mcm2-7 interact through the Mcm4–Orc1 and Mcm7–Orc1 interfaces with additional stabilizing contacts between the Mcm3 and Mcm6 WHDs with Cdc6 and Orc4–Orc5 subunits (Fig. 5*A*). The global tilt of the ORC–Cdc6 results in the DNA outside the ORC central cavity becoming wedged between Orc5-6 and Mcm2. Importantly, the transition of DNA from the bent to the straight conformation occurs gradually through several discrete intermediates (Fig. 5*A*), in which the DNA contacts to Mcm2 outcompete the interactions with the outer surface of ORC. This competition observed in the MEP is made possible by the flexible nature of the ORC–Cdc6–Mcm2-7 interface, which primarily involves the highly mobile WHDs of the Mcm2-7 (Fig. 5*A* and Movie S1).

The optimized MEP also depicts the expected conformational switching of Mcm2-7 from a spiral to a planar ring. Mcm2-7 loading onto DNA proceeds through DNA insertion through the gap created by the Mcm2 and Mcm5 subunits (18, 42). For the pre-insertion OCCM, the narrow gap in the spiral conformation of Mcm2-7 precludes direct insertion of the DNA duplex. Thus, DNA threading through the opening between Mcm2 and Mcm5 is an active process, which has been captured in detail by the chain-of-replicas MD simulation (Fig. 5*D* and Movie S1). Notably, the Mcm5, Mcm3, and Mcm7 subunits undergo a considerable upward shift toward ORC–Cdc6 prior to DNA insertion, while much smaller relative displacements are observed for Mcm2, Mcm6, and Mcm4. These structural changes lead to a widening of the Mcm2–Mcm5 interface during DNA insertion by 15 Å. Finally, our optimized path depicts the transition of the Mcm4 WHD from the inner to the outer side of the complex as observed in the published OCCM structure (28). The switch in WHD positioning occurs in the latter stages of the conformational transition and is facilitated by the global tilt of ORC–Cdc6, which creates an opening between Orc1 and Mcm7 that allows the passage of the WHD through the newly formed gap (Fig. 5*E*).

### PCA Analysis Captures 10 On-Path DNA Insertion Intermediates.

**Fig. 6.**
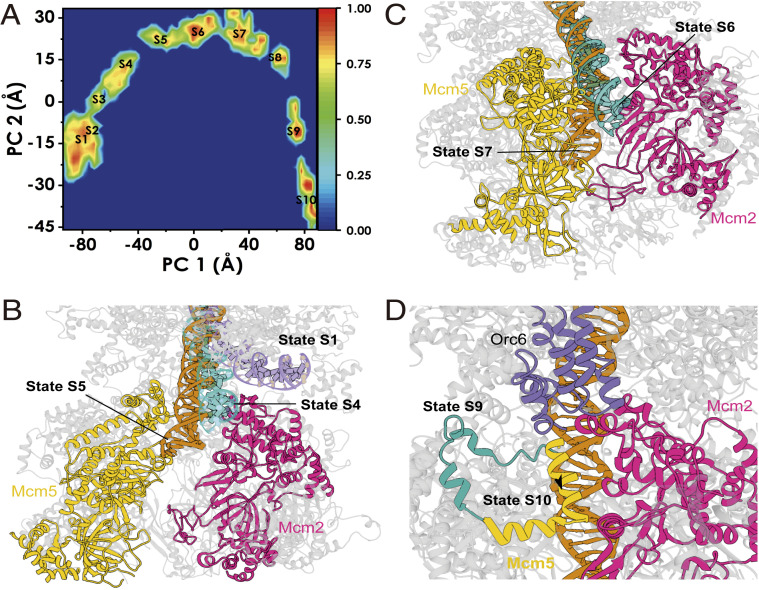
The 10 computationally captured structural intermediates (S1 to S10) of Mcm2-7 during loading onto DNA by ORC–Cdc6. (*A*) Normalized two-dimensional histograms of principal component 1 (PC 1) versus principal component 2 (PC 2). Centroids from clustering are labeled S1 to S10. (*B*) Early intermediates (states S1 to S5) depict ORC-induced repositioning of the dsDNA near the Mcm2 and Mcm5 loading gate. Multiple states for the dsDNA (pre-insertion OCCM, state S4 and state S5) are shown and colored. (*C*) dsDNA entry and passage through the Mcm2 and Mcm5 loading gate (states S6 and S7). Multiple states for the dsDNA are labeled and colored. (*D*) Mcm5 gate closing helix moves across the gate to form an interface with Mcm2, signaling the end of loading.

To more extensively sample the conformational ensemble along the optimal energy path, we further performed free MD simulations of all on-path intermediates. Releasing all string replicas from the imposition of spatial restraints allowed the simulation trajectories to sample in minima of the underlying free energy landscape. We then carried out principal component analysis (PCA) on the combined trajectories, which is well suited to separate the observed conformational states and identify the predominant motions that the protein complex adopt (47). Fig. 6*A* shows the histogram of our trajectory data projected onto the first two principal components (PC1 and PC2), representing the two largest motions involved in the transition: PC1 is capturing the global tilt and twist motion of ORC–Cdc6 with respect to the Mcm2-7 core (Movie S2), and PC2 is capturing the gate opening and closing motion of the Mcm2 and Mcm5, along with an out-of-plane motion of the Mcm2-7 ring (Movie S3). Our sampling covered the entire path from the pre-insertion OCCM to the gate-closed OCCM. Minima on the PC1 vs. PC2 histogram corresponded to discerned intermediates whose structures can be extracted for further analysis. Thus, we performed an agglomerative clustering analysis to subdivide our conformational ensemble into 10 consecutive on-path intermediary states (S1 to S10) and extracted centroid conformations from each cluster to analyze residue-level contacts (Fig. 6 *B*–*D* and Movie S4).

#### Preinsertion intermediates (S1 to S4).

States 1 to 3 (S1 to S3) correspond to early intermediates in the Mcm2-7 loading transition, in which the DNA remains stably bound to ORC–Cdc6. While maintaining a stable hold on the DNA, ORC–Cdc6 twists counterclockwise with respect to Mcm2-7 and partially tilts toward Mcm2. Importantly, state S4 captures an intermediate wherein the DNA is flanked by and makes contacts with Orc5, Orc6, and Mcm2. The pinched DNA conformation between ORC–Cdc6 and Mcm2 exemplifies the competition for dsDNA binding that eventually releases the Orc6 grip on the DNA. In addition to global tilting, states S1 to S4 display the beginning of the Mcm2-7 transition toward a more planar conformation with a wider opening between the Mcm2 and Mcm5 subunits. Such planar ring conformations have been observed in low-resolution structures for *Encephalitozoon cuniculi*, *Drosophila melanogaster*, and *Saccharomyces cerevisiae* MCM hexamers (11, 48, 49). Observing the out-of-plane motion of the Mcm2-7 early in the overall transition toward the OCCM conformations implies that the helicase is flexible in its open conformation and the motion is relatively facile.

#### Gate-passage intermediates (S5 to S7).

Cluster S5 reveals a preinsertion intermediate with the DNA positioned directly above the open Mcm2–Mcm5 gate and interacting with both Mcm2 and Mcm5. Mcm4 WHD, found inside the hexamer channel, binds and bends the DNA by ∼35 °, propping up the duplex toward Mcm2. Thus, clusters S5, S6, and S7 represent intermediates along the passage of the DNA duplex through the Mcm2-7 gate and into the hexamer inner cavity. From state S5 to S7, the Mcm2-7 gate widens up by ∼15 Å, which is sufficient to accommodate the lateral dimension of dsDNA. The inner surfaces of the Mcm2 and Mcm5 AAA+ domains are lined with positively charged and polar residues, providing stabilizing interactions for the DNA. Specific contacts with Mcm gate residues appear to facilitate the passage of the DNA (*SI Appendix*, Fig. S5). Notably, a helix of Mcm5 (Ile526–Lys534) was found to insert into the major groove, forming direct DNA contacts (e.g., Mcm5 Arg529 inserting into the groove, Mcm5 Lys534 with the DNA backbone). The transition of the dsDNA through the Mcm2–Mcm5 gate is accompanied by gradual straightening of the DNA helical axis and is stabilized by DNA contacts with Orc5, Mcm4 WHD, Mcm2 AAA+ domain, and Mcm5. DNA insertion is also accompanied by Mcm5 WHD rearrangement. Interactions between the Mcm5 and Mcm4 WHDs help to position the Mcm4 WHD to contact the DNA.

#### Gate-closure intermediates (S8 to S10).

Upon passing the gate (after state S7), DNA encounters positively charged residues on the interior surface of the Mcm2-7 channel. Indeed, we and others have suggested that the strong negative charge of the DNA contributes to closure of the Mcm2-7 ring (12, 18, 42). Specifically, Mcm3 and Mcm6 establish multiple contacts with the DNA. Insertion of the Mcm5 helix (Ile526–Lys534) into the DNA helix persists through state S8 but is then fully removed in state S9, thus serving only a transient role in stabilizing the DNA along its passage through the Mcm2-7 gate. In states S8 and S9, Mcm2 and Mcm5 come together to partially close the gate proximal to the Mcm2 AAA+ domain. In state S8, the Mcm4 WHD still maintains contact with the DNA. However, in state S9, this domain shifts toward the Mcm7–Orc1 interface and completely breaks contact with the duplex and the Mcm5 WHD. In the initial pre-insertion OCCM conformation, Mcm4 WHD occupies space within the Mcm2-7 inner cavity, while, in the final gate-closed OCCM conformation, the WHD has positioned on the outside. Therefore, our minimum energy path unavoidably has to reposition the Mcm4 WHD from the inside to the outside of the complex. Furthermore, Mcm4 WHD needs to move out to complete the tilting motion of the ORC–Cdc6 (Orc5 and Orc6 have to bind to Mcm2 and Mcm5 in the gate-closed OCCM) and achieve complete straightening of the DNA passing through the central channel of Mcm2-7. In our minimum free energy path, this conformational switch of the Mcm4 WHD occurs in the very last stages of the Mcm2-7 loading process (clusters S8, S9, and S10). Tilting of the ORC–Cdc6 with respect to the Mcm2-7 core by an additional ∼12 ° frees up sufficient space to allow passage of Mcm4 WHD through the gap between Orc1 and Mcm7. This indicates that flexible linkage of ORC/Cdc6 and Cdt1/Mcm2-7 is important to facilitate the transition of WHDs from a central location via a gap between the two complexes to a distal location, where the WHDs do not interfere with DNA insertion. Another notable conformational change occurring from state S9 to S10 is the repositioning of a gate bridging helix of Mcm5 (residues Glu-563 to Thr-577), which binds Mcm2 to stabilize the closed conformation of the Mcm2–Mcm5 interface in the gate-closed OCCM state.

## Summary

**Fig. 7.**
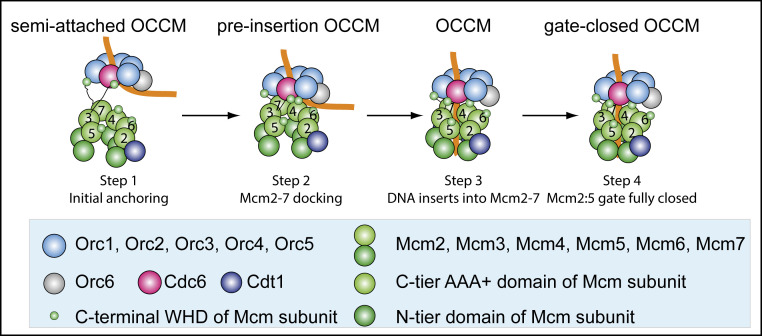
Summary of four key molecular events during the loading of the first Cdt1-bound Mcm2-7 hexamer by ORC–Cdc6. Step 1: the Mcm3 and Mcm7 WHDs make the first contact with ORC–Cdc6 in the semi-attached OCCM. At this stage, Mcm2-7 is only flexibly linked to ORC–Cdc6 via the “double anchor” of Mcm3 and Mcm7 WHDs. Step 2: additional contacts are made between ORC–Cdc6 and Cdt1–Mcm2-7 in the pre-insertion OCCM, positioning DNA near the Mcm2–Mcm5 gate. Mcm2-7 is still a left-handed open spiral at this stage. Step 3: Cdt1–Orc4–Mcm6 (AAA+) interactions restructure the complex as observed in both mutant OCCM and the WT OCCM structures. DNA is inserted in the complex, and the complex assumes a ring structure with a small gap at the Mcm2–Mcm5 N-terminal interface. Step 4: closure of the Mcm2-7 ring at the N-terminal interface, as observed in the WT gate-closed OCCM.

Our work reveals two conformations of the OCCM complex prior to DNA insertion. The semi-attached OCCM and the pre-insertion OCCM, together with the mutant OCCM observed here and the WT OCCM published earlier, as well as the gate-closed OCCM (18), reveals a four-step DNA loading process of Mcm2-7 by ORC–Cdc6 (Fig. 7). The semi-attached OCCM is characterized by high flexibility between ORC–Cdc6 and Cdt1–Mcm2-7. This indicates that the Mcm3 and Mcm7 WHDs act as anchor points, capturing ORC–Cdc6. Moreover, these interactions guarantee that Cdt1–Mcm2-7 recognizes the ORC–Cdc6 complex but not ORC alone, since both WHDs contact Cdc6, thus providing complex specificity. This is consistent with the observation that a Mcm3–Mcm5 subcomplex can interact with ORC–Cdc6. However, the extreme Mcm3 C terminus, which is essential for Orc–Cdc6 interaction (15), was not visible in our structure, probably due to flexibility. *S. cerevisiae* ORC–Cdc6 bends DNA, similarly as seen in 2D class averages of the *Drosophila* ORC–Cdc6–DNA complex (34) and the high-resolution structure of the budding yeast ORC–DNA complex (33). The pre-insertion OCCM is characterized by additional ORC–Cdc6–Cdt1–Mcm2-7 interactions. Excitingly, we directly observed that ORC–Cdc6 positions the DNA close to the open Mcm2–Mcm5 DNA entry gate of Cdt1–Mcm2-7. Thus, our data identify a key OCCM intermediate prior to DNA insertion, which was previously only predicted to exist (33, 50). The lack of an Mcm2 WHD supports the direct positioning of DNA on the Mcm2 AAA+ domain, as observed in the pre-insertion OCCM. Thus, our structure provides a molecular explanation why only budding yeast Mcm2 lacks a WHD, as this configuration directly supports helicase loading. However, higher eukaryotes contain a small Mcm2 WHD, where it might help to position DNA near the gate through a series of conserved basic residues. Our molecular simulation reveals a series of motions associated with DNA insertion that are inaccessible by experimental means and discovered an Mcm5 helix at the Mcm2–Mcm5 interface that plays a transient role to facilitate DNA insertion.

## Materials and Methods

### Protein complex assembly from purified proteins.

The *S. cerevisiae* loading intermediate mutant OCCM, which is missing the Mcm6 C-terminal extension (amino acids 839 to 1017), was assembled in vitro with purified ORC, Cdc6, Cdt1, and mutant Mcm2-7 on plasmid DNA containing the ARS1 sequence in the presence of ATPγS and isolated via a previously described magnetic bead pull-down approach (51), with minor modifications. Twelve pre-RC reactions containing 40 nM ORC, 80 nM Cdc6, 40 nM Cdt1, 40 nM mutant Mcm2-7, and 6 nM pUC19-ARS1 beads in 50 μL of buffer A (50 mM Hepes-KOH, pH 7.5, 100 mM potassium glutamate, 10 mM magnesium acetate, 50 μM zinc acetate, 3 mM ATPγS, 5 mM DTT, 0.1% Triton X-100, and 5% glycerol) were incubated for 15 min at 24 °C. After three washes with buffer B (50 mM Hepes-KOH, pH 7.5, 100 mM K acetate, 10 mM magnesium acetate, 3 mM ATPγS), the complex was eluted with 1 U of DNase I in buffer B and 1 mM CaCl_2_ for 5 min.

### Cryo-Grid Preparation.

To prepare cryo-EM grids, we applied 3 μL of the loading reaction sample at a final concentration of 0.7 mg/mL to glow-discharged C-flat 1.2/1/3 holey carbon grids, incubated for 10 s at 6 °C and 95% humidity, blotted for 3 s, then plunged into liquid ethane using an FEI Vitrobot IV. We loaded the grids into an FEI Titan Krios electron microscope operated at 300-kV high tension and collected images semiautomatically with SerialEM under low-dose mode at a magnification of ×22,500 and a pixel size of 1.31 Å per pixel. A Gatan K2 Summit direct electron detector was used under superresolution mode for image recording with an under-focus range from 1.5 to 3.5 μm. The dose rate was 10 electrons per Å^2^ per second, and the total exposure time was 9 s. The total dose was divided into a 30-frame movie, and each frame was exposed for 0.3 s.

### Image Processing and 3D Reconstruction.

Approximately 10,000 raw movie micrographs were collected. The movie frames were first aligned and superimposed by the program Motioncorr (52). Contrast transfer function parameters of each aligned micrograph were calculated using the program CTFFIND4 (53). All of the remaining steps, including particle auto selection, 2D classification, 3D classification, 3D refinement, and density map postprocessing were performed using Relion-2.0 (54). We manually picked ∼10,000 particles from different views to generate 2D class averages, which were used as templates for subsequent automatic particle selection. Automatic particle selection was then performed for the entire data set. A total of 838,544 particles were initially selected. Particles were then sorted by similarity to the 2D references; about 10% of particles with the lowest *z*-scores were deleted from the particle pool. Two-dimensional classification of all remaining particles was performed, and particles in unrecognizable classes were removed. The remaining “good” particles were divided into four subsets according to 2D averages: (*i*) mutant OCCM, (*ii*) semi-attached OCCM, (*iii*) semi-attached OCCM in which Orc-Cdc6-DNA density is well resolved but most Mcm2-7 density as well as Cdt1 were missing, and (*i*v) open spiral mutant Mcm2-7 hexamer. Three-dimensional classification was performed separately for each subset, leading to four 3D models. The particles belonging to each 3D model were used for further 3D refinement, resulting in the 4.3-Å average resolution cryo-EM 3D map of semi-attached OCCM, the 7.7-Å average resolution cryo-EM 3D map of the mutant Mcm2-7 hexamer, the 8.1-Å average resolution cryo-EM 3D map of the pre-insertion OCCM, and the 10.5-Å average resolution cryo-EM 3D map of the mutant OCCM. The resolution of these maps was estimated by the gold-standard Fourier shell correlation at the correlation cutoff value of 0.143. The 3D density maps were corrected for the detector modulation transfer function and sharpened by applying a negative B-factor.

### Structural Modeling, Refinement, and Validation.

The model building of the 4.3-Å 3D map of semi-attached OCCM and the 8.1-Å 3D map of the pre-insertion OCCM was based on the published structures of *S. cerevisiae* OCCM (referred to as WT OCCM in the current manuscript; PDB IF code 5V8F) and ORC-DNA (PDB ID code 5ZR1). For modeling of the semi-attached OCCM 3D map at 4.3-Å resolution, Chimera was used to first rigid-body dock the isolated Cdc6, Mcm3 WHD, and Mcm7 WHD model extracted from the WT OCCM (PDB ID code 5V8F) and the ORC-DNA model (PDB ID code 5ZR1) into the corresponding EM map, and then each Orc subunit, Cdc6, Mcm3 WHD, and Mcm7 WHD, were further individually and manually fitted as a rigid-body in COOT (55). The DNA structure was also manually adjusted and extended using COOT. The obtained model was then refined in real space against the cryo-EM 3D map using the phenix.real_space_refine module in PHENIX (56). Finally, the quality of the refined atomic models was examined using MolProbity (57). For modeling of the pre-insertion OCCM map at 8.1 Å, the atomic models of Mcm2-7 and ORC–Cdc6–DNA were extracted separately from the WT OCCM and were directly docked as rigid bodies into the EM map in Chimera (58). The initial rigid-body docking was followed by manual adjustment using COOT (55). Due to the low resolution and per the community custom, the manually adjusted atomic model was not subject to further refinement. We did not build atomic models for the 3D maps of the “mutant Mcm2-7” hexamer and the “mutant OCCM” because these mutant structures were similar to the corresponding WT structures that had been reported (18, 33). Structural figures were prepared in Chimera and PyMOL (https://pymol.org/2/).

### Computational Modeling.

We constructed models of pre-insertion OCCM and the gate-closed OCCM complex. We then used molecular dynamics flexible fitting (MDFF) (59) to refine the models into the respective EM density maps. Prior to MDFF, each model was solvated with equilibrated TIP3P solvent in a simulation box with 15 Å spacing from the protein complex to the edge of the box. Counterions, Na^+^ and Cl^−^, were added to neutralize the overall charge of the complex and adjust salt concentration to 150 mM. The solvated systems were then minimized for 10,000 steps, heated in the NVT ensemble, and then equilibrated in the NPT ensemble (1 atm and 300 K). During equilibration, positional restraints on all heavy atoms were gradually reduced from 5 to 0 kcal mol^−1^ Å^−2^ while simultaneously employing MDFF grid forces with a scaling factor of 0.1. We then guided the equilibrated structures of the pre-insertion OCCM into the gate-closed OCCM configuration using targeted molecular dynamics (TMD). The TMD production run was completed in 20 ns and employed 1,000 kcal/mol force constant on the backbone atoms of the protein and DNA. Detailed procedures are described in *SI Appendix, Supporting Information Methods*. The initial MCM-loading path was represented by 49 evenly spaced snapshots taken from the targeted MD trajectory. This path was then optimized using the finite-temperature string method with swarms of trajectories. String method protocol and definition of collective variables are provided in the *SI Appendix*. The optimized configurations were then released for 50 ns of free unbiased MD per replica. This resulted in ∼2.5 μs of aggregate simulation time. The combined trajectories were then subject to principal component analysis and agglomerative clustering with CPPTRAJ (60). VMD and UCSF Chimera packages were used for analysis and visualization (58, 61).

## Supplementary Material

Supplementary File

Supplementary File

Supplementary File

Supplementary File

Supplementary File

## Data Availability

The 3D cryo-EM maps of semi-attached OCCM (4.3 Å), pre-insertion OCCM (8.1 Å), mutant OCCM (10.5 Å), and mutant Mcm2-7 hexamer (7.7 Å) have been deposited in the Electron Microscopy Data Bank under accession codes EMDB-21662, EMDB-21665, EMDB-21666, and EMDB-21664, respectively. The atomic models of semi-attached OCCM, pre-insertion OCCM, mutant OCCM, and mutant Mcm2-7 hexamer have been deposited in the Protein Data Bank under accession codes 6WGC, 6WGG, 6WGI, and 6WGF, respectively.
