# Supplementary material for: Structural mechanism of helicase loading onto replication origin DNA by ORC-Cdc6
Source: Proc Natl Acad Sci U S A. 2020 Jul 15;117(30):17747–56. doi: 10.1073/pnas.2006231117 (PMC7395460; doi:10.1073/pnas.2006231117)
Supplement: Supplementary File [file pnas.2006231117.sapp.pdf]

PNAS
www.pnas.org

Supplementary Information for

# Structural mechanism of helicase loading onto replication origin DNA by ORC-Cdc6

Zuanning Yuan, Sarah Schneider, Thomas Dodd, Alberto Riera, Lin Bai, Chunli Yan, Indiana Magdalou, Ivaylo Ivanov, Bruce Stillman, Huilin Li, and Christian Speck

**Corresponding authors:** Ivaylo Ivanov, Bruce Stillman, Huilin Li, and Christian Speck

**Email:** Stillman@cshl.edu, IIvanov@gsu.edu, Huilin.Li@vai.org, or Chris.Speck@imperial.ac.uk

**This PDF file includes:**

Tables S1
Figures S1 to S5
Legends for Movies S1 to S4

**Other supplementary materials for this manuscript include the following:**

Movies S1 to S4



www.pnas.org/cgi/doi/10.1073/pnas.2006231117

**Table S1.** Cryo-EM data collection, 3D reconstruction, and refinement statistics of the four OCCM intermediates containing Mcm6 C-terminal WHD deletion.

| **Data Collection** | "semi-attached OCCM" | "pre-insertion OCCM" | "mutant OCCM" | "mutant Mcm2-7" hexamer |
|---|---|---|---|---|
| Database ID | EMDB-21662<br>PDB ID 6WGC | EMDB-21665<br>PDB ID 6WGG | EMDB-21666<br>PDB ID 6WGI | EMDB-21664<br>PDB ID 6WGF |
| EM equipment | TFS Titan Krios | TFS Titan Krios | TFS Titan Krios | TFS Titan Krios |
| Voltage (kV) | 300 | 300 | 300 | 300 |
| Detector | Gatan K2 | Gatan K2 | Gatan K2 | Gatan K2 |
| Pixel size (Å) | 1.31 | 1.31 | 1.31 | 1.31 |
| Electron dose ($e^-$/Å$^2$) | 50 | 50 | 50 | 50 |
| Defocus range (µm) | 1.5 - 3.5 | 1.5 - 3.5 | 1.5 - 3.5 | 1.5 - 3.5 |
| | | | | |
| **3D reconstruction** | | | | |
| Number of used particles | 86,074 | 25,126 | 27,830 | 24,072 |
| Resolution (Å) | **4.3** | **8.1** | **10.5** | **7.7** |
| Sharpening B-factor (Å$^2$) | -166 | -34 | -149 | -322 |
| **Model composition** | | | | |
| Peptide chains | 9 | 14 | 14 | 6 |
| Protein residues | 2592 | 6635 | 6632 | 3638 |
| **R.m.s deviations** | | | | |
| Bonds length (Å) | 0.004 | | | |
| Bonds Angle | 0.95° | | | |
| **Ramachandran plot** | | | | |
| Preferred (%) | 92.62 | | | |
| Allowed (%) | 7.27 | | | |
| Outlier (%) | 0.11 | | | |
| **Validation** | | | | |
| Molprobity score | 1.95 (99%) | | | |
| Rotamer outliers (%) | 0.04 | | | |

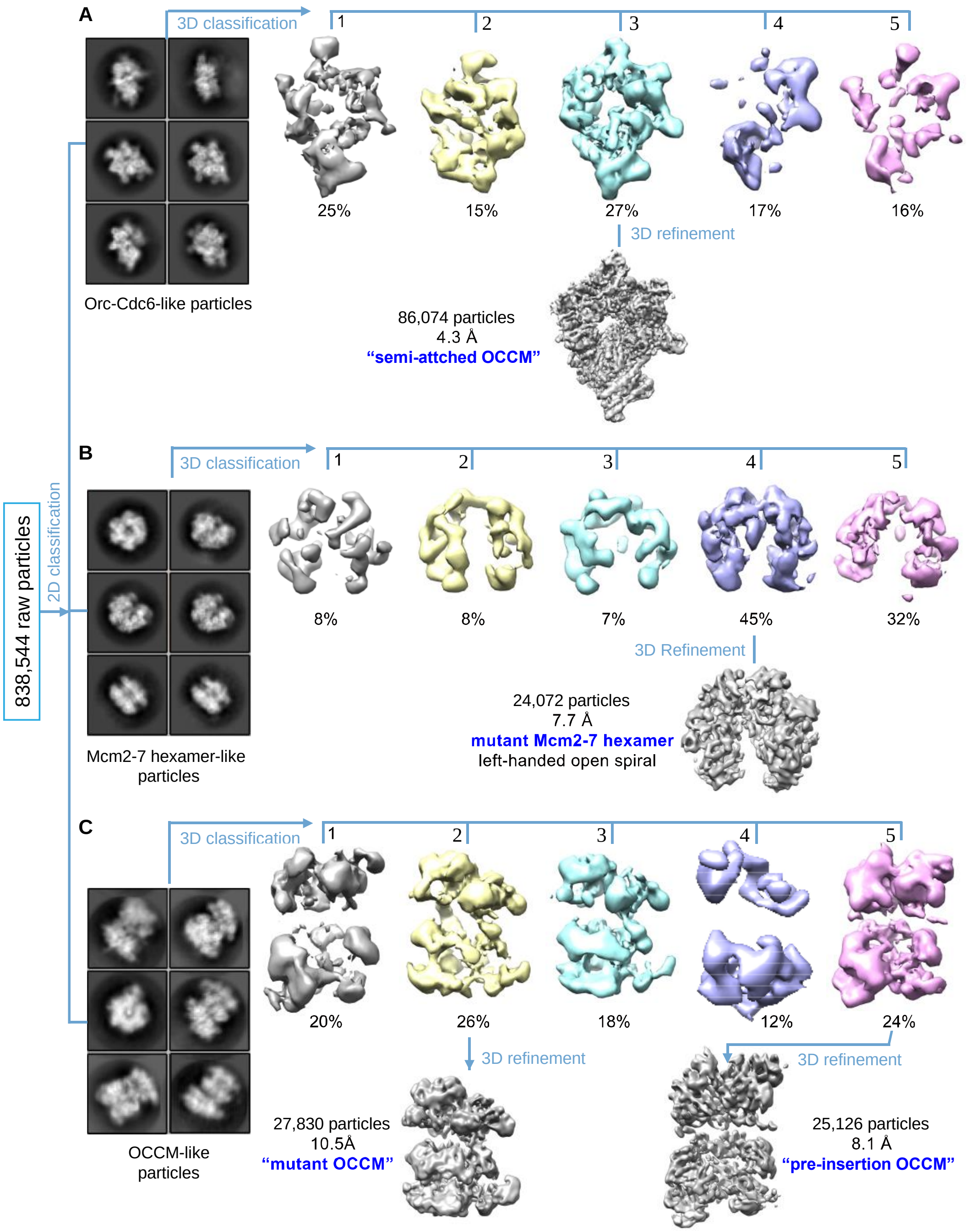


**Fig. S1.** Image processing and 3D reconstruction of cryo-EM micrographs of the loading reaction mixture of ORC, Cdc6, Cdt1, and Mcm2-7 containing Mcm6ΔC6. After 2D classification, particles are sorted into three categories as shown in panels A-C. (**A**) ORC-Cdc6-like particles whose further 3D classification and refinement led to the "semi-attached OCCM" in which the main body of Mcm2-7 was flexible and lack density except for the Mcm3 and Mcm7 WHDs. (**B**) Mcm2-7 hexamer like particles whose further 3D classification and refinement led to the 7.7 Å resolution 3D map of the left-handed open spiral structure of "mutant Mcm2-7". (**C**) OCCM-like particles in which both ORC-Cdc6 and Cdt1-Mcm2-7 densities were present. After further 3D classification and refinement, two structures were obtained – the "OCCM" at 10.5 Å resolution and the "pre-insertion OCCM" at 8.1 Å resolution.

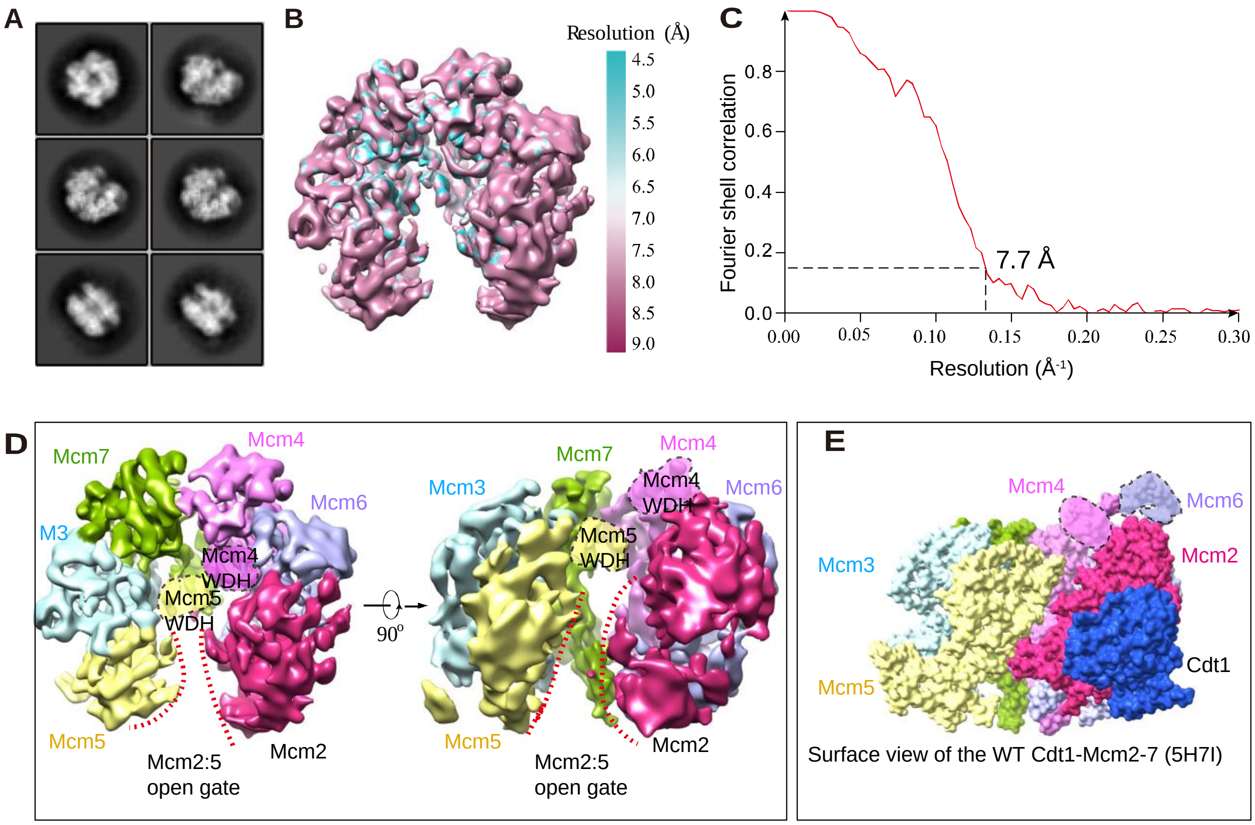


**Figure S2.** 3D reconstruction of the “mutant Mcm2-7” hexamer. **(A)** 2D class averages of hexamer-like particles. **(B)** Local resolution map. **(C)** Gold-standard Fourier shell correlation curves indicating an average resolution of 7.7 Å. (**D**) 3D map of “mutant Mcm2-7” hexamer in top and side views. Note the presence of WHDs of Mcm3, Mcm4, and Mcm5 but the lack of the Mcm6 WHD due to the deletion. (**E**) A side view of the WT Cdt1 bound Mcm2-7 (PDB ID 5H7I) showing the presence of Mcm6 WHD.

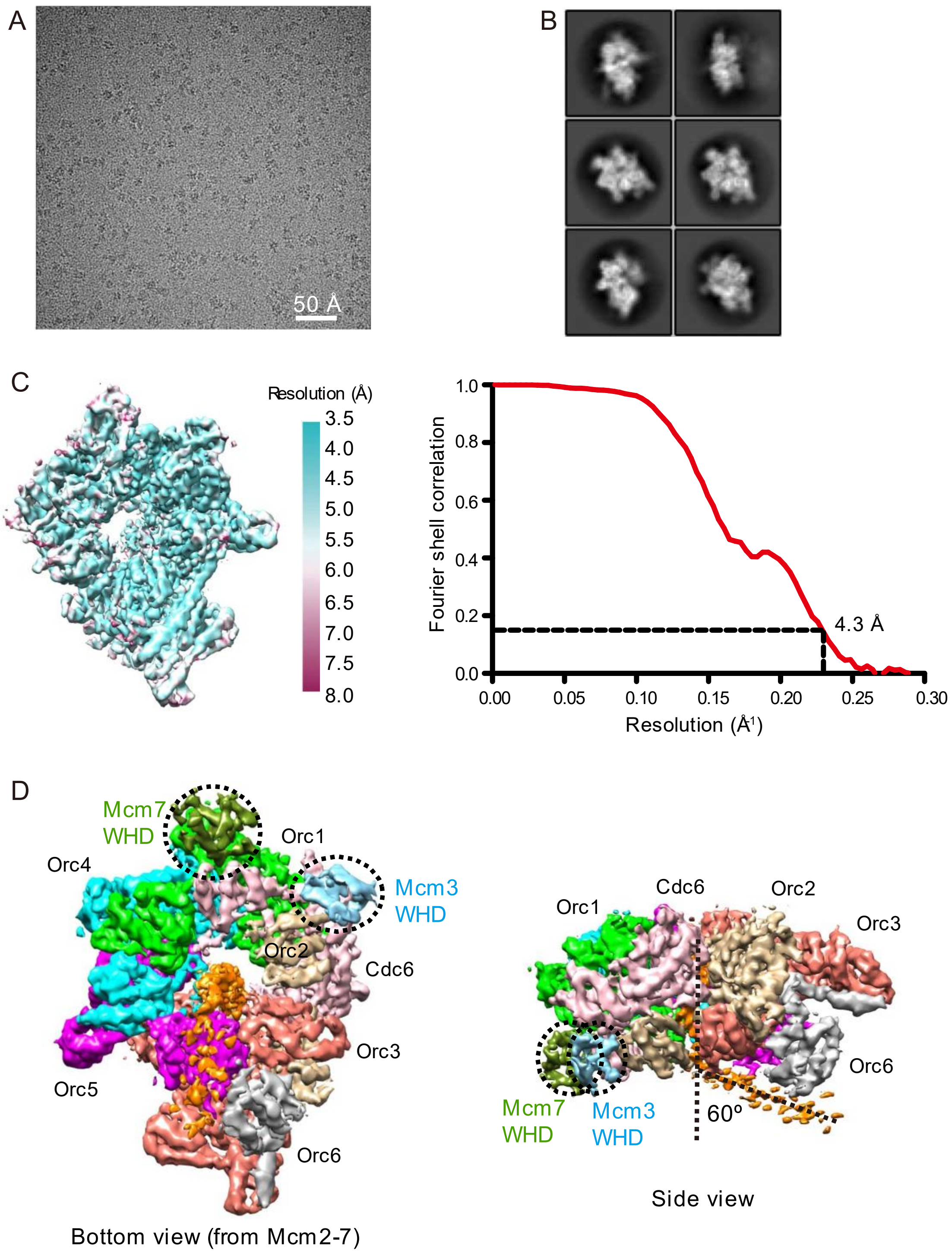


**Figure S3.** 3D reconstruction of the "semi-attached OCCM" structure. **(A)** A typical raw micrograph of the loading reaction mixture. **(B)** 2D class averages of the ORC-Cdc6-like particles. **(C)** Local resolution map (left) and the Gold standard Fourier shell correlation curves (right) with the FSC cutoff value 0.143 indicated. (**D**) Bottom (left) and side (right) views of the surface-rendered 3D map of the "semi-attached OCCM" with individually colored subunits. The presence of the Mcm3 and Mcm7 WHDs are highlighted by two dashed black circles. The two black lines approximately trace the dsDNA density, indicating an approximately 60[illegible] bend as the DNA emerges from the central channel of ORC-Cdc6 and binds to the positively charged C-tier surface.

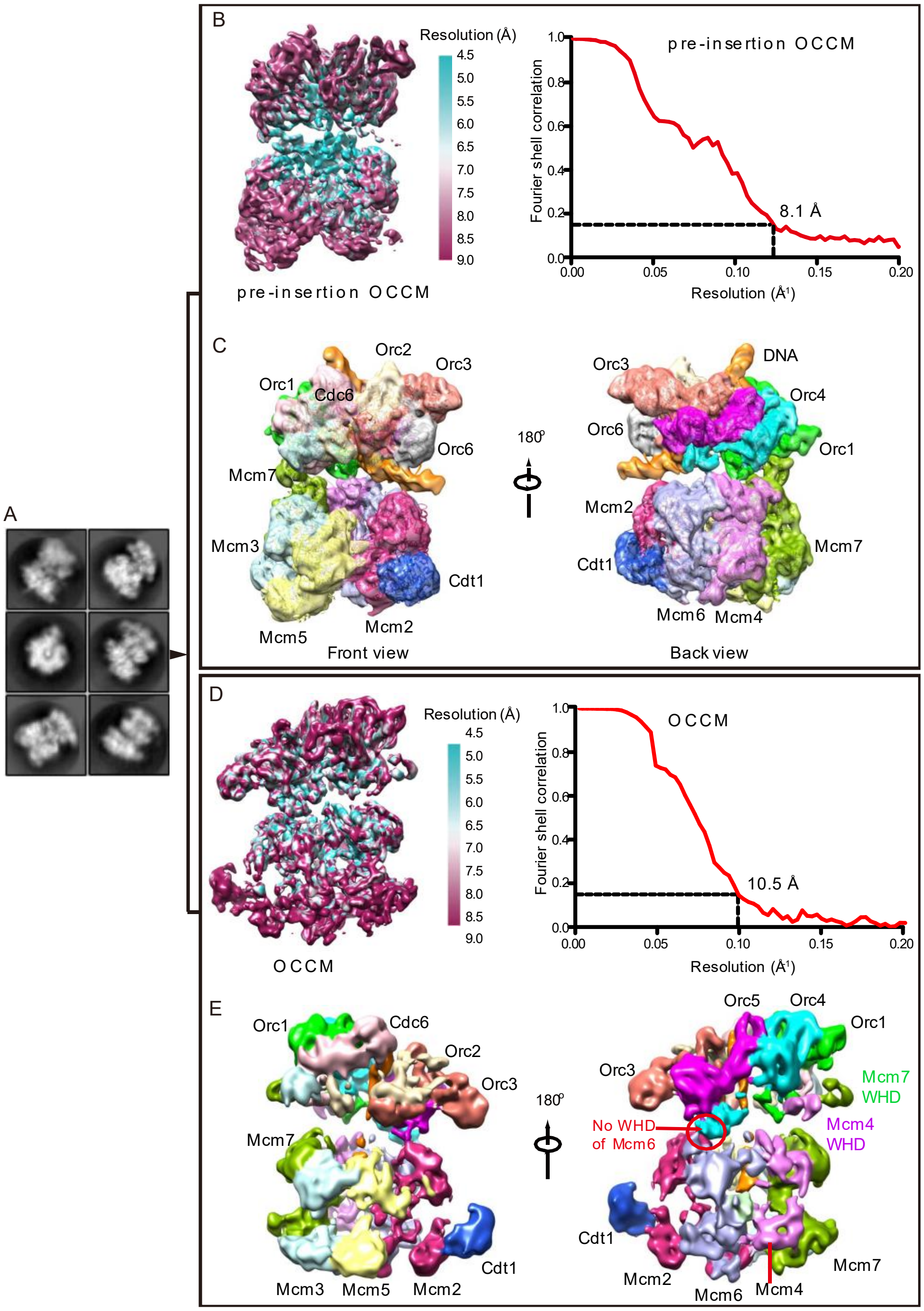


**Figure S4.** 3D classification and reconstruction of the OCCM-like particles led to the identification of "OCCM" and "pre-insertion OCCM". **(A)** 2D class averages of the "OCCM-like" particles. **(B)** Local resolution map (left) and the Fourier shell correlation curves (right) of the "pre-insertion OCCM" structure at 8.1 Å average resolution. **(C)** Front and back side views of the 3D map of the "pre-insertion OCCM" structure. The three major contact sites between ORC-Cdc6 and Cdt1-Mcm2-7 are marked by three dashed black circles. (**D**) Local resolution map (left) and the Fourier shell correlation curve (right) of the 10.5 Å resolution 3D map of the "mutant OCCM" structure. (**E**) Front and back side views of the "mutant OCCM" 3D map with subunits individually colored. Note the absence of the Mcm6 WHD due to the deletion in this mutant complex, yet the structure is otherwise highly similar to the previously published WT "OCCM" structure.

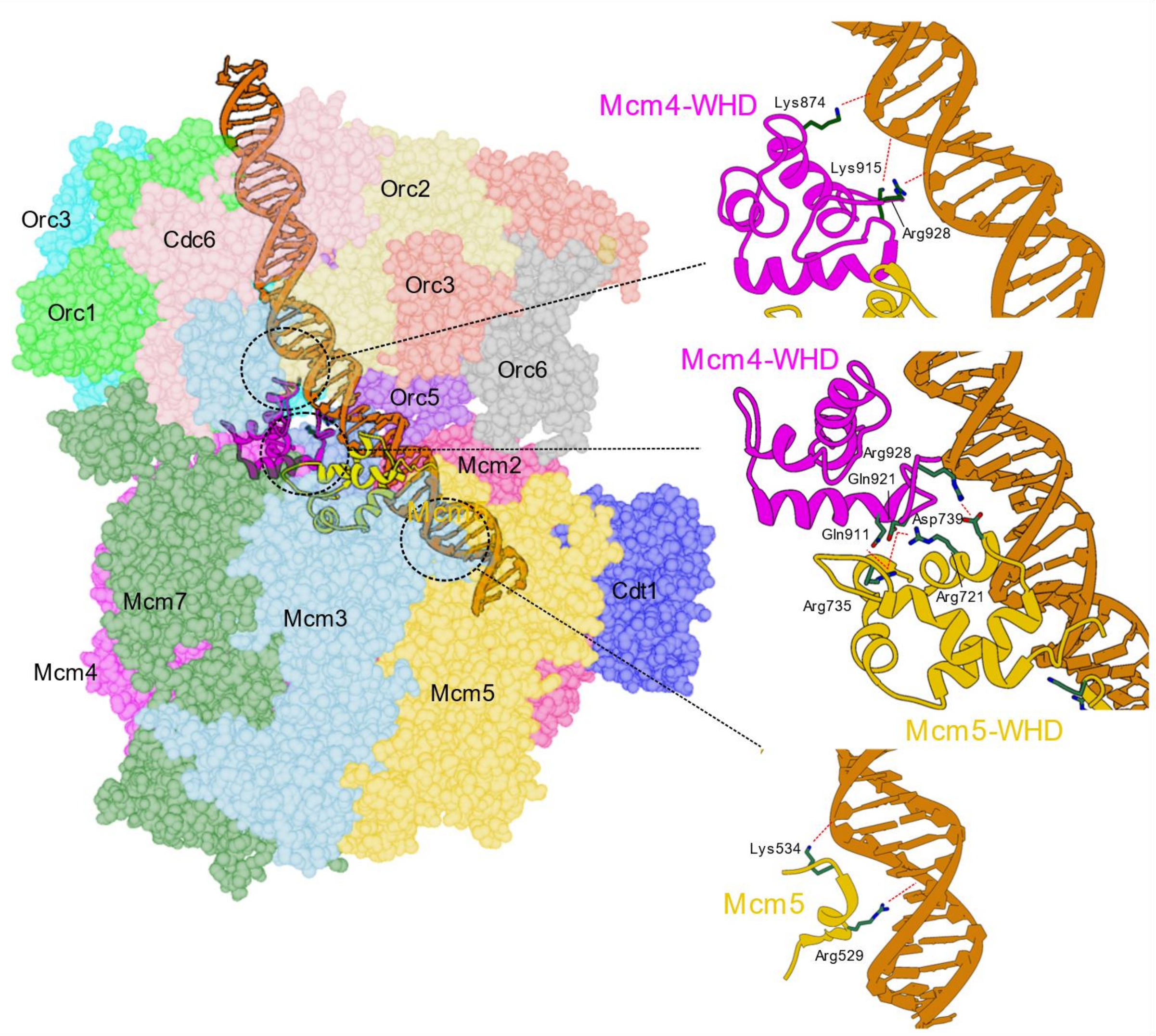


**Figure S5. Multiple interactions stabilize dsDNA as it loaded through the Mcm2-Mcm5 gate.** Mcm4-WHD stabilizes the bent DNA through electrostatic interactions with the negatively charged backbone of DNA. Positioning of Mcm4-WHD is mediated through contacts with Mcm5-WHD. The insertion helix of Mcm5 (Mcm5-IH) stabilizes downstream DNA as it passes through the Mcm2/Mcm5 gate. All protein subunits for both ORC-Cdc6 and Mcm2-7-Cdt1 are labeled and colored.

**Movie S1 (separate file).** "pre-insertion OCCM" to "gate-closed OCCM" transition optimized with the finite-temperature string method with swarms of trajectories.

**Movie S2 (separate file).** Global tilt and twist of ORC-Cdc6 with respect to Mcm2-7. Principal component 1 (PC1) captures the global tilt and twist of ORC-Cdc6.

**Movie S3 (separate file).** Opening and closing of the Mcm2-Mcm5 gate. Principal component 2 (PC2) captures the opening of the loading gate to allow the passage of dsDNA and subsequent closing once dsDNA reaches the central channel of Mcm2-7 hexamer.

**Movie S4 (separate file).** Conformational intermediates identified through clustering analysis of the molecular dynamics' trajectories. Electrostatic interactions between Orc1-6, Mcm2-7 and dsDNA facilitate the passage of the dsDNA through the loading gate. Residue-level contacts are shown as spheres. Near the end of the transition, the Mcm4-WHD passes through the opening created by the Orc1-6 and Mcm2-7. State S9 captures the Mcm4-WHD wedged between the Orc1-6 and Mcm2-7.
